# Fialuridine Induces Acute Liver Failure in Chimeric TK-NOG Mice: A Model for Detecting Hepatic Drug Toxicity Prior to Human Testing

**DOI:** 10.1371/journal.pmed.1001628

**Published:** 2014-04-15

**Authors:** Dan Xu, Toshi Nishimura, Sachiko Nishimura, Haili Zhang, Ming Zheng, Ying-Ying Guo, Marylin Masek, Sara A. Michie, Jeffrey Glenn, Gary Peltz

**Affiliations:** 1 Department of Anesthesia, Stanford University School of Medicine, Stanford, California, United States of America; 2 Central Institute for Experimental Animals, Kawasaki, Japan; 3 Center for the Advancement of Health and Biosciences, Sunnyvale, California, United States of America; 4 Department of Drug Disposition, Eli Lilly and Company, Indianapolis, Indiana, United States of America; 5 Department of Medicine, Stanford University School of Medicine, Stanford California, United States of America; 6 Department of Microbiology and Immunology, Stanford University School of Medicine, Stanford California, United States of America; 7 Department of Pathology, Stanford University School of Medicine, Stanford California, United States of America; GlaxoSmithKline, United Kingdom

## Abstract

**Background:**

Seven of 15 clinical trial participants treated with a nucleoside analogue (fialuridine [FIAU]) developed acute liver failure. Five treated participants died, and two required a liver transplant. Preclinical toxicology studies in mice, rats, dogs, and primates did not provide any indication that FIAU would be hepatotoxic in humans. Therefore, we investigated whether FIAU-induced liver toxicity could be detected in chimeric TK-NOG mice with humanized livers.

**Methods and Findings:**

Control and chimeric TK-NOG mice with humanized livers were treated orally with FIAU 400, 100, 25, or 2.5 mg/kg/d. The response to drug treatment was evaluated by measuring plasma lactate and liver enzymes, by assessing liver histology, and by electron microscopy. After treatment with FIAU 400 mg/kg/d for 4 d, chimeric mice developed clinical and serologic evidence of liver failure and lactic acidosis. Analysis of liver tissue revealed steatosis in regions with human, but not mouse, hepatocytes. Electron micrographs revealed lipid and mitochondrial abnormalities in the human hepatocytes in FIAU-treated chimeric mice. Dose-dependent liver toxicity was detected in chimeric mice treated with FIAU 100, 25, or 2.5 mg/kg/d for 14 d. Liver toxicity did not develop in control mice that were treated with the same FIAU doses for 14 d. In contrast, treatment with another nucleotide analogue (sofosbuvir 440 or 44 mg/kg/d po) for 14 d, which did not cause liver toxicity in human trial participants, did not cause liver toxicity in mice with humanized livers.

**Conclusions:**

FIAU-induced liver toxicity could be readily detected using chimeric TK-NOG mice with humanized livers, even when the mice were treated with a FIAU dose that was only 10-fold above the dose used in human participants. The clinical features, laboratory abnormalities, liver histology, and ultra-structural changes observed in FIAU-treated chimeric mice mirrored those of FIAU-treated human participants. The use of chimeric mice in preclinical toxicology studies could improve the safety of candidate medications selected for testing in human participants.

*Please see later in the article for the Editors' Summary*

## Abstract 2

Gary Peltz, Jeffrey Glenn, and colleagues report that a pre-clinical mouse toxicology model can detect liver toxicity of a drug that caused liver failure in several early clinical trial participants in 1993.

*Please see later in the article for the Editors' Summary*

## Editors' Summary

**Background:**

Before new drugs are approved for clinical use, they undergo extensive preclinical (laboratory-based) and clinical testing. In the preclinical studies, scientists investigate the causes of diseases, identify potential new drugs, and test promising drug candidates in animals. Animal testing is performed to determine whether the new drug is likely to work, and to screen for drug-induced toxicity. In preclinical toxicology studies, new drugs are given to two or more animal species to find out whether the drug has any short- or long-term toxic effects such as damage to the liver (hepatotoxicity). Drugs that pass these animal tests enter clinical trials. Phase I clinical trials test new drugs in a handful of healthy volunteers or patients to evaluate their safety and to identify possible side effects. In phase II trials, a larger group of patients receives the new drug to evaluate its safety further and to get an initial idea of its effectiveness. Finally, in phase III trials, very large groups of patients are randomly assigned to receive the new drug or an established treatment for their disease. These randomized controlled trials provide detailed information about the effectiveness and safety of a candidate drug, and must be completed before a drug can be approved for clinical use.

**Why Was This Study Done?:**

Since animals are not perfect models for people, candidate drugs can cause toxicities in clinical trials that were not predicted by preclinical toxicology testing performed using animal species. For example, in 1993, 15 participants in a phase II trial were given a nucleoside analogue called fialuridine to treat hepatitis B virus infection (nucleoside analogues often have antiviral activity). Seven participants developed liver failure and lactic acidosis (buildup of lactic acid in the blood). Analysis of liver tissue from the affected participants revealed steatosis (fatty degeneration), intracellular fat droplets, and swollen mitochondria (these organelles are the powerhouses of the cell). Five participants subsequently died, and two had to have a liver transplant. In preclinical toxicology testing in mice, rats, dogs, and primates, there had been no indications that fialuridine would be hepatotoxic in people. It now seems that the expression of a nucleoside transporter in the mitochondria of humans but not of other animals may underlie the human-specific mitochondrial toxicity and hepatotoxicity of fialuridine. With several other nucleoside analogues in development, a better screening tool for human-specific mitochondrial toxicity is needed. In this study, the researchers investigate whether fialuridine toxicity can be detected in TK-NOG mice with chimeric (humanized) livers. TK-NOG mice are immunodeficient mice that have been genetically engineered so that human liver cells (hepatocytes) transplanted into these animals establish a long-lived mature “human organ.”

**What Did the Researchers Do and Find?:**

The researchers treated chimeric (with transplanted human liver cells) and control (without transplanted human liver cells) TK-NOG mice with several doses of fialuridine. After treatment with the highest dose (1,600-fold above the dose used in the phase II trial) for four days, the chimeric mice developed liver failure and lactic acidosis. Moreover, steatosis and lipid and mitochondrial abnormalities developed in the regions of their livers that contained human hepatocytes but not in regions that contained mouse hepatocytes. Notably, the control mice had not developed liver toxicity after 14 days of treatment with the highest dose of drug. Liver toxicity was also easily detectable in chimeric mice that had been treated for 14 days with a fialuridine dose only 10-fold above that used in the human trial. Treatment with another nucleoside analogue that does not cause liver toxicity in people did not cause liver toxicity in the chimeric mice.

**What Do These Findings Mean?:**

These findings show that fialuridine-induced liver toxicity can be readily detected using TK-NOG mice that have humanized livers at drug doses only 10-fold higher than those that caused liver failure in the phase II trial. Although the liver toxicity developed much more quickly in these mice than in the human trial participants, the clinical features, laboratory abnormalities, and structural changes seen in the fialuridine-treated chimeric TK-NOG mice closely mirrored those seen in fialuridine-treated people. The use of TK-NOG mice containing humanized livers in toxicology testing will not reveal whether drugs have human-specific toxicities outside the liver. Since they are highly immunocompromised, chimeric TK-NOG mice cannot be used to detect immune-mediated drug toxicities. Nevertheless, these findings suggest that the use of chimeric mice in toxicology studies could help improve the safety of candidate drugs that are tested in humans.

**Additional Information:**

Please access these websites via the online version of this summary at http://dx.doi.org/10.1371/journal.pmed.1001628.

- The US Food and Drug Administration, the body that approves drugs for clinical use in the US, provides an overview for patients about the drug development process from the laboratory to the clinic
- The UK Medicines and Healthcare Products Regulatory Agency (MHRA) provides more detailed information for patients and the public about the drug development process, including a section on preclinical research, which includes information on animal testing
- The US National Institutes of Health provides information about clinical trials, including personal stories from people who have taken part in clinical trials
- The UK National Health Service Choices website has information for patients about clinical trials and medical research, including personal stories about participation in clinical trials
- Understanding Animal Research is a UK advocacy group that provides information about the importance of animal research to the public, teachers, scientists, journalists, and policy makers
- Wikipedia has a page on animal testing (note that Wikipedia is a free online encyclopedia that anyone can edit; available in several languages)

## Introduction

In 1993, a phase II clinical trial with 15 participants was performed to evaluate the safety and efficacy of a 6-mo course of a nucleoside analogue (fialuridine [FIAU] 0.1 or 0.25 mg/kg/d) for hepatitis B virus infection [1]. The results during the first 8 wk of treatment were promising; there was a substantial virological response, and very few adverse events were reported. However, the study was terminated by week 13 because of serious unanticipated complications. Seven participants developed hepatic failure, lactic acidosis, and pancreatic failure; five of these participants died, and two required emergency liver transplants. Histological analysis of liver tissue revealed steatosis and cholestasis, and electron microscopy revealed cytoplasmic fat droplets and swollen mitochondria that contained a reduced number of cristae [1]. Some evidence indicates that expression of a nucleoside transporter in human (but not in mouse) mitochondria may be responsible for the human-specific mitochondrial toxicity caused by FIAU [2]. While there was no evidence of toxicity in five participants who received a cumulative dose of ≤200 mg (treated for less than 4 wk), the cumulative FIAU dose in participants developing hepatic failure ranged from 551 to 1,753 mg, and the toxicity progressed after discontinuation of therapy.

A National Academy of Sciences retrospective analysis of all preclinical FIAU toxicity data, which included acute and chronic dose studies performed in multiple laboratory animals (mice, rats, dogs, and monkeys), concluded that the available animal toxicity data provided no indication that FIAU treatment would cause such tragic consequences in human participants [3]. In these studies, dose-dependent toxicities occurred at or near the maximum tolerated dose in each animal species tested. In particular, dogs (3 mg/kg/d for 90 d), monkeys (25 mg/kg/d for 30 d), rats (500 mg/kg/d IV for 30 d), and mice (50 mg/kg/d for 90 d) did not show significant histological or biochemical abnormalities. Only four of 50 mice treated with 250 mg/kg/d (∼1,000-fold above the maximum human dose) died of renal failure. In a study performed after the human trial, the drug was well tolerated in rats treated for 10 wk with a dose of FIAU (510 mg/kg/d) that is 2,000-fold above the maximum human dose; the treated rats had normal alanine aminotransferase (ALT) and aspartate aminotransferase levels, but had increased lactate levels [3].

This striking example indicates that inadequate screening tools for human-specific drug toxicities can have tragic consequences. Although many recently approved antivirals are nucleoside analogues, this class of drugs has been associated with toxicities appearing in clinical trials, which led to a discontinuation of their development (e.g., balapiravir [4], BMS-986094, and IDX-184). The fact that drug-induced toxicity was recognized only during clinical testing is a concern, especially since multiple other nucleoside analogues are nearing approval (e.g., sofosbuvir [5],[6]) or are in development (e.g., mericitabine [7]). Since we lack methods to predict human-specific mitochondrial toxicities, these considerations are also relevant for other drug classes.

Chimeric mice with humanized livers have been produced to generate a more predictive preclinical platform, and they can mediate human-specific drug biotransformation reactions (reviewed in [8]). However, it has not been determined whether chimeric mice can be used to predict human-specific drug toxicities. To overcome the limitations inherent in prior chimeric mouse models, the TK-NOG mouse [9] was produced by expressing a herpes simplex virus type 1 thymidine kinase (TK) transgene within the liver of a highly immunodeficient mouse strain (NOG) [10]. A brief exposure to a nontoxic dose of ganciclovir causes the rapid and temporally controlled ablation of mouse liver cells expressing the transgene. The absence of ongoing liver toxicity enables transplanted human liver cells to develop into a mature “human organ” with a three-dimensional architecture and gene expression pattern characteristic of mature human liver, which can be stably maintained for >6 mo without exogenous drug treatments [9]. Chimeric TK-NOG mice have been used to predict the pattern of human drug metabolism, to investigate the occurrence of a human drug–drug interaction (prior to human exposure) for a drug in development [11], to identify human genetic factors affecting the metabolism of important drugs [12], and to produce a novel method for autologous human liver regeneration using adipocyte stem cells [13]. Since TK-NOG mice do not have ongoing liver toxicity, and do not require treatment with other drugs to suppress their immune system or to prevent liver damage [8], they could provide an optimal platform for toxicology studies. Therefore, we investigated whether toxicology studies using chimeric TK-NOG mice could have predicted the occurrence of FIAU-induced liver failure in humans.

## Methods

### Preparation and Characterization of Chimeric TK-NOG Mice

All animal experiments were performed according to protocols approved by the Stanford Institutional Animal Care and Use Committee. The results are reported according to the ARRIVE guidelines [14]. TK-NOG mice were obtained from In Vivo Sciences International. Chimeric TK-NOG mice with humanized livers were prepared as previously described [12]. Cryopreserved human hepatocytes were obtained from Celsis In Vitro. Data on the chimeric mice, the hepatocyte donors, and the level of human serum albumin are shown in Table S1. All chimeric mice used in this study had a human serum albumin level greater than 6.5 mg/ml. Human liver cells were transplanted when the mice were 8 wk old, and the toxicology studies were performed 8 wk after transplantation. The plasma human albumin level, which is linearly correlated with the extent of liver humanization, was measured by radioimmunoassay as described previously [9]. The plasma lactate levels were measured using an Accutrend Plus Lactate Analyzer (Heska) according to the manufacturer's instructions.

### Toxicology Studies

FIAU was obtained from Eli Lilly; it was shown to be >99% pure by liquid chromatography–mass spectrometry analysis, and its chemical structure was confirmed by 2-D nuclear magnetic resonance analysis. For formulation, FIAU powder was first dissolved in DMSO to a concentration of 500 mg/ml, and then diluted to a working solution of 50 mg/ml with saline. TK-NOG mice with non-humanized (control) or humanized livers were treated with 2.5, 25, 100, or 400 mg/kg/d of FIAU by oral gavage for 4 to 14 d. Sofosbuvir was obtained from Nanosyn; it was >99% pure by high-performance liquid chromatography analysis. For formulation, sofosbuvir was first dissolved in DMSO to a concentration of 250 mg/ml, and then diluted to working solutions of 40 mg/ml and 6 mg/ml with saline. Control TK-NOG mice or TK-NOG mice with humanized livers were treated with Sofosbuvir (44 or 440 mg/kg/d) by oral gavage for 14 d. For FIAU and sofosbuvir, separate groups of mice with humanized livers were simultaneously treated with vehicle (5% DMSO).

Liver tissue was harvested and placed in 10% formalin for histology and immunohistology, and in 4% glutaraldehyde for transmission electron microscopy (TEM) analyses. For oil red O staining, frozen sections were fixed in cold acetone for 10 min prior to staining. For analysis by TEM, liver tissue was fixed in 2% paraformaldehyde/2.5% glutaraldehyde in a 0.1 M sodium cacodylate buffer solution for 24 h. The samples were then post-fixed in 2% osmium tetroxide, dehydrated, and embedded in LX 112 epon resin (Thermo Fischer Scientific, NC9925769). Thin sections were prepared, then stained with uranyl acetate and lead citrate, and then viewed using a Hitachi H7650 electron microscope. Adjacent liver sections were stained with toluidine blue to identify the regions containing murine or human cells, which were analyzed by TEM.

### Immunohistology

Sections of formalin-fixed paraffin-embedded liver were stained with hematoxylin and eosin (H/E) or with monoclonal antibody to human cytokeratin 8. For immunohistochemistry, the slides were de-paraffinized (xylene, 2×10 min; 100% ethanol, 2×10 min; and 95% ethanol, 5 min), rinsed in water, and rehydrated in 1 mM EDTA buffer (pH 9.0). The slides were then boiled in 1 mM EDTA buffer (pH 9.0) in a microwave oven for 10 min for heat-induced antigen retrieval. The slides were then sequentially incubated with a 1∶100 dilution in PBS of mouse monoclonal antibody to human cytokeratin 8 (clone M20, catalog number ab9023, Abcam) or an isotype-matched negative control monoclonal antibody (Pharmingen) for 1 h at room temperature, with second- and third-stage antibodies per manufacturer's instructions (MACH 4 Universal HRP-Polymer Kit, Biocare Medical), and then with Betazoid DAB solution (Biocare Medical). There were three washes in PBS after each incubation step. The slides were counterstained with hematoxylin, dehydrated with graded ethanol and xylene solutions, and placed on a cover slip for evaluation by a pathologist (S. A. M.) who did not have any knowledge of the drug treatment given to each mouse. Immunoperoxidase-stained slides were evaluated by light microscopy for the location of the DAB (brown) on the slides stained with the anti–human cytokeratin 8 monoclonal antibody, and compared to the isotype-matched negative control monoclonal antibody. H/E-stained slides were evaluated by light microscopy, and the location of murine versus human tissue was determined by comparison to serial sections that were stained with anti–human cytokeratin 8 monoclonal antibody. The slides were photographed with a Nikon Microphot FXA microscope with plan apochromat objectives (4×/0.10 NA, 10×/0.25, 20×/0.40, 40×/0.65, and 60×/0.95), a SPOT Insight Color Mosaic camera (model 14.2, Diagnostic Instruments), and SPOT Advanced imaging software (version 4.6).

### Statistical Analyses

The correlation between the measured lactate and human serum albumin levels was determined using the Pearson's correlation with a correction for the effect of the two different human hepatocyte donors (Table S1). This correction factor was calculated using the CORR procedure in SAS (version 9.3), with the donor set as the confounding variable. To statistically assess the measured differences in ALT and lactate measured on days 0 and 4, the difference in the log-transformed ALT (or lactate) levels between days 4 and 0 was calculated. It was observed that the means of this difference among mice transplanted with liver cells from the two donors were not significant (*p* = 0.49 and 0.35 for ALT and lactate, respectively), and neither were the variances (Bartlett's test of homogeneity of variances, *p* = 0.85 and 0.82, respectively) [15]. Therefore, the data obtained from mice prepared from the two donors could be pooled to assess whether the observed difference between days 4 and 0 was significant. A paired-sample *t* test was then applied to assess whether the ALT and lactate levels were different on days 4 and 0. A two-sample *t* test was used to assess whether the ALT and lactate levels were different between control TK-NOG mice and TK-NOG mice with humanized livers. To represent the range of ALT and lactate levels measured in the different groups of mice analyzed, we present the mean ± standard deviation throughout this article.

## Results

### FIAU-Induced Liver Toxicity Was Observed in TK-NOG Mice with Humanized Livers

**Figure 1:**
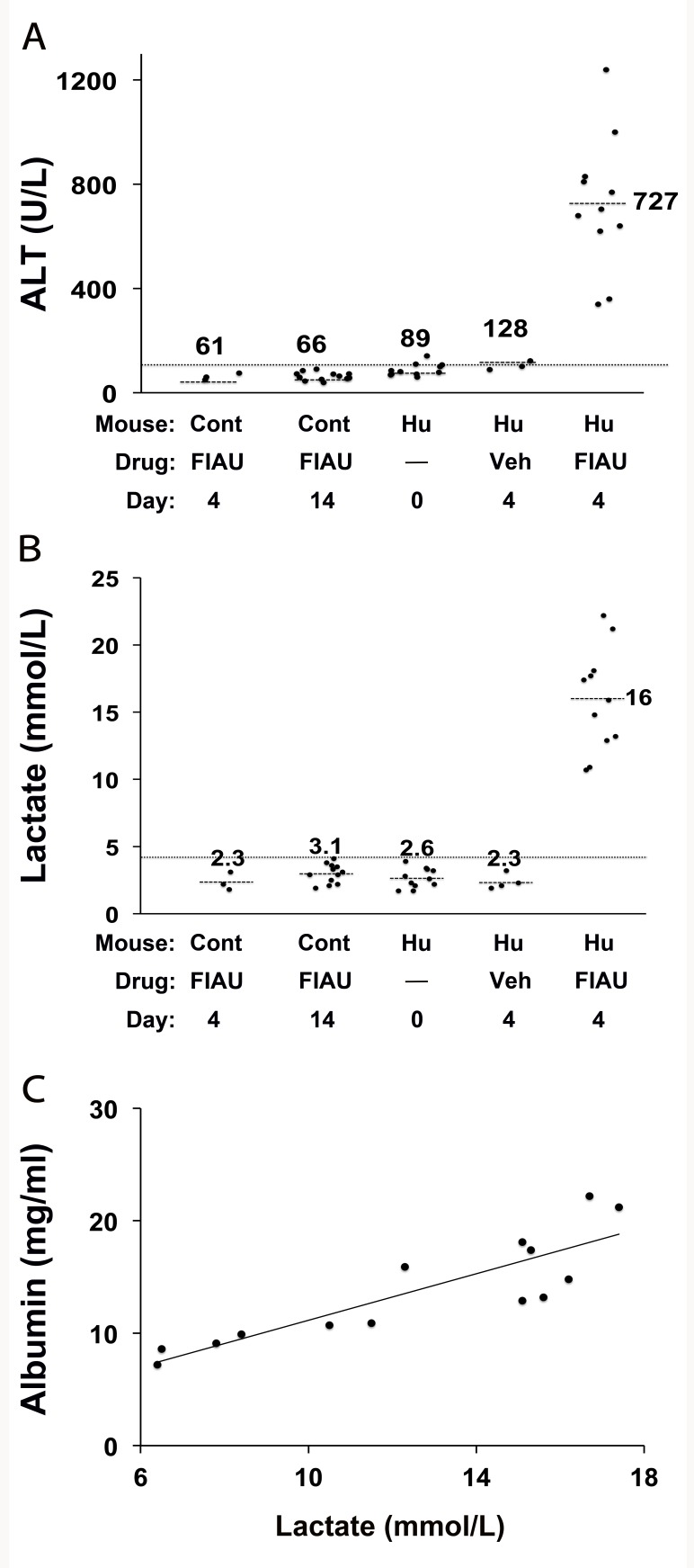
FIAU-induced liver toxicity develops in TK-NOG mice with humanized livers. Control (non-humanized) (Cont, *n* = 12 per group) or chimeric (Hu, *n* = 15 per group) TK-NOG mice were treated with 400 mg/kg/d FIAU or vehicle (Veh) for 0, 4, or 14 d, and the plasma ALT (A) and lactate (B) levels were measured on the indicated days. The lactate and ALT levels were elevated in FIAU-treated chimeric mice, while control mice had normal ALT and lactate levels after 14 d of FIAU dosing. Each symbol represents the value measured in a control TK-NOG mouse or a TK-NOG mouse with a humanized liver; the dotted line across the graph indicates the upper limit of normal, and the short dashed lines show the average for each group. (C) The correlation between the human albumin and plasma lactate levels measured in 15 chimeric TK-NOG mice after 4 d of treatment with 400 mg/kg/d FIAU. The human serum albumin is a measure of the extent of liver humanization in chimeric TK-NOG mice. Each dot represents the values measured in one chimeric TK-NOG mouse.

Consistent with prior toxicology results, control (non-humanized) TK-NOG mice treated with a high dose of FIAU (400 mg/kg/d po) for 14 d tolerated the drug, and all treated control mice had normal plasma lactate (3.0 mmol/l±0.8) and ALT (63.7 U/l±15.7) levels (Figure 1A and 1B). However, chimeric TK-NOG mice with a high level (average of 12.5 mg/ml plasma human albumin, corresponding to >90%) of liver humanization (Table S1) could not tolerate treatment with this dose of FIAU. By the third day, all treated chimeric mice were lethargic, and several were overtly jaundiced. Since one mouse died and the other 14 remained extremely lethargic, drug treatment was terminated on day 4. Consistent with their appearance, all FIAU-treated chimeric mice had elevated serum lactate (15.9 mmol/l±3.8) and ALT (726 U/l±257.5) levels, which were significantly above their pretreatment values (*p* = 5×10^−8^ and 1×10^−7^, respectively). The lactate and ALT values were 5-fold and 11-fold, respectively, above those in control (non-humanized) TK-NOG mice treated with the same dose of FIAU for 4 or 14 d (Figure 1A and 1B). In contrast to the FIAU-treated chimeric mice, vehicle-treated chimeric mice had a normal appearance, and normal plasma ALT and lactate levels (Figure 1A and 1B). Consistent with FIAU causing a human-specific toxicity, the degree of lactate elevation correlated with the extent of liver humanization (Pearson's correlation: 0.86), and this correlation was highly significant (*p*<0.0001) (Figure 1C).

**Figure 2:**
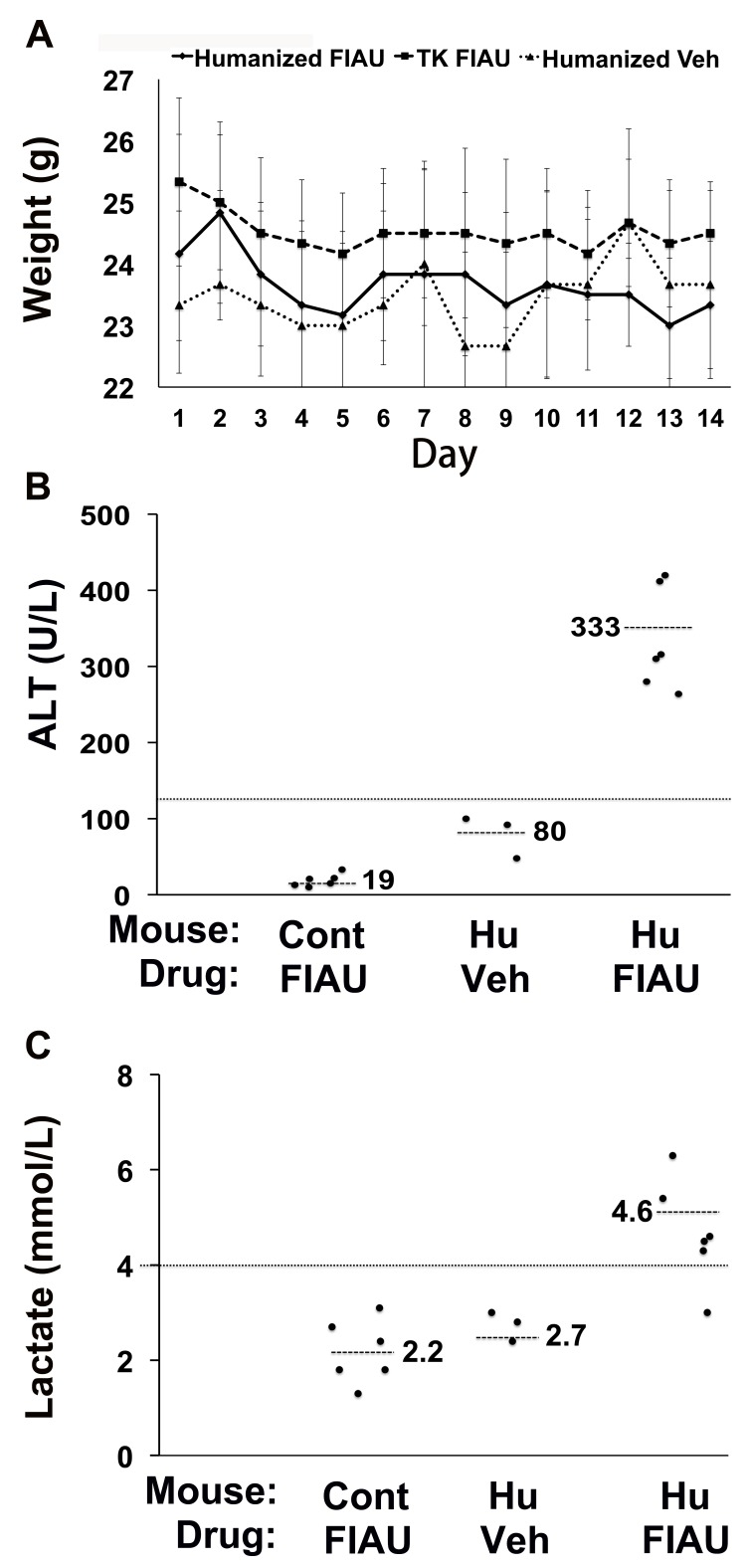
Liver toxicity develops in TK-NOG mice with humanized livers treated with 25 mg/kg/d FIAU. Control (non-humanized) (TK or Cont, *n* = 6 per group) or chimeric (Hu, *n* = 6 per group) TK-NOG mice were treated with 25 mg/kg/d FIAU or vehicle (Veh) for 14 d, and their body weights (A) and plasma ALT (B) and lactate (C) levels were measured on the indicated days. (A) There was no difference in the weights of vehicle- or FIAU-treated control TK-NOG mice or TK-NOG mice with humanized livers. In (B and C), each symbol represents the value measured in a control TK-NOG mouse or a TK-NOG mouse with a humanized liver, the dotted line across the graph indicates the upper limit of normal, and the short dashed lines show the average for each group.

**Figure 3:**
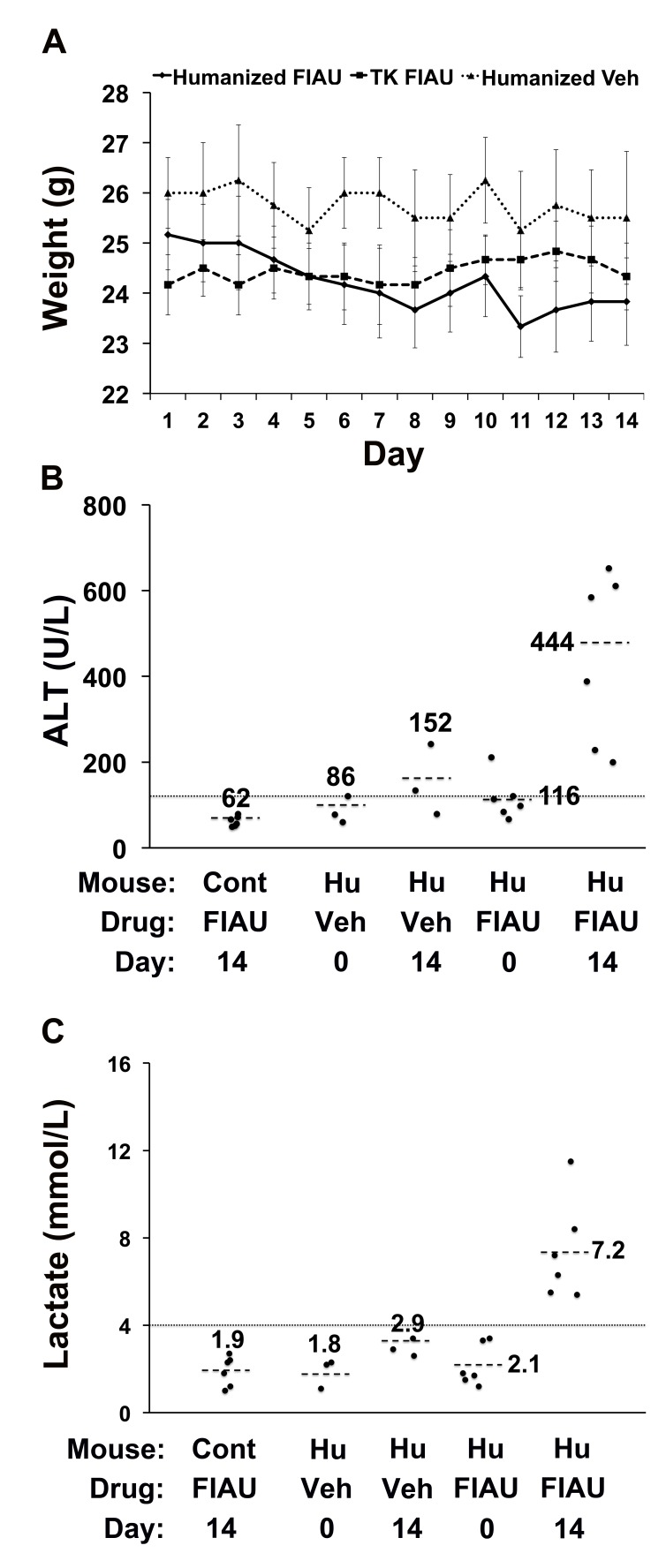
FIAU-induced liver toxicity is dose-dependent in TK-NOG mice with humanized livers. Control (non-humanized) (TK or Cont, *n* = 6 per group) or chimeric (Hu, *n* = 6 per group) TK-NOG mice were treated with 2.5 mg/kg/d FIAU or vehicle (Veh) for 14 d, and their body weights (A) and plasma ALT (B) and lactate (C) levels were measured on the indicated days. (A) There was no difference in the weights of vehicle- or FIAU-treated control TK-NOG mice or TK-NOG mice with humanized livers. In (B and C), each symbol represents the value measured in a control TK-NOG mouse or a TK-NOG mouse with a humanized liver, the dotted line across the graph indicates the upper limit of normal, and the short dashed lines show the average for each group.

To investigate whether this toxicity was dose-dependent, chimeric TK-NOG mice with highly humanized livers (Table S1) were then treated with FIAU 100 mg/kg/d po. The 100-mg/kg/d dosing regimen was better tolerated than a 400-mg/kg/d dose; the chimeric mice were treated for 17 d before dosing was electively terminated because of weight loss. Chimeric TK-NOG mice lost weight over the 17-d treatment period, while FIAU-treated control TK-NOG mice did not (*p* = 0.028) (Figure S1). However, there were no deaths, and none of the FIAU-treated chimeric mice were overtly jaundiced. However, there was clear serological evidence of liver injury in the FIAU-treated mice with humanized livers; their serum ALT (235 U/l±72) was significantly increased (*p* = 0.0008, over 4-fold) relative to FIAU- (63 U/l±17) or vehicle-treated (60 U/l±7) control mice (Figure S1). Of importance, the serum ALT was not elevated in FIAU-treated relative to vehicle-treated control TK-NOG mice (Figure S1). Mice with humanized livers were next treated with FIAU 25 mg/kg/d po for 14 d, before the dosing was electively terminated. Although there were no deaths and the FIAU-treated mice with humanized livers did not lose body weight (Figure 2A), there was clear evidence of drug-induced liver toxicity. The FIAU-treated mice with humanized livers had statistically significant elevations of plasma transaminase (*p* = 0.0001; Figure 2B) and serum lactate (*p* = 0.013; Figure 2C) levels relative to vehicle-treated mice with humanized livers. Liver toxicity also developed in TK-NOG mice with humanized livers that were treated with a 10-fold lower FIAU dose (2.5 mg/kg/d) for 14 d. Although the mice with humanized livers treated with this dose of FIAU did not lose weight (*p* = 0.24) relative to vehicle-treated mice with humanized livers (Figure 3A), they had statistically significant elevations of plasma transaminase (*p* = 0.047; Figure 3B) and serum lactate (*p* = 0.008; Figure 3C) levels relative to vehicle-treated mice with humanized livers. These data establish that FIAU-induced liver toxicity could be detected in chimeric TK-NOG mice, and this dose-dependent toxicity could easily be detected at FIAU doses as low as 10-fold above the dose used in humans.

**Figure 4:**
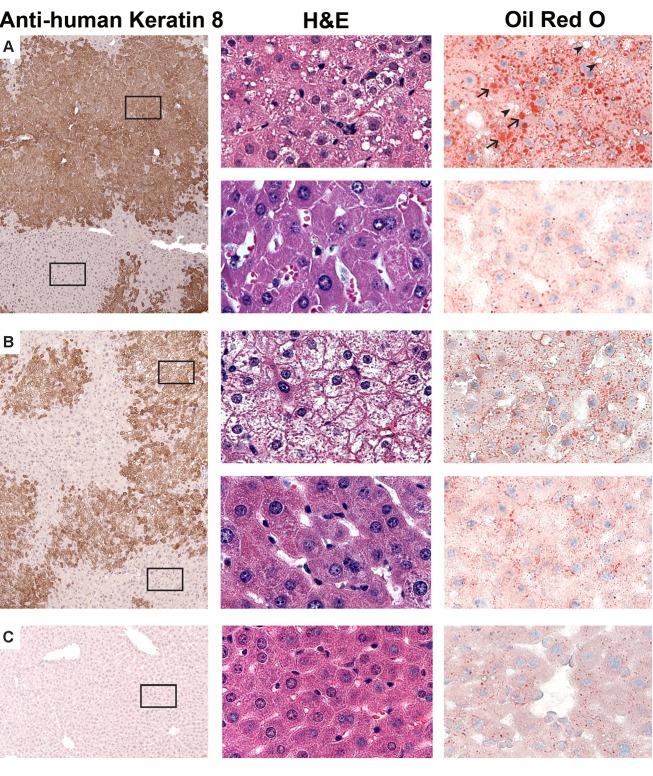
Histopathology of FIAU-induced liver toxicity in TK-NOG mice with humanized livers. Liver tissues were obtained from mice with humanized livers treated with FIAU 400/kg/d (#192) (A) or FIAU 0 mg/kg/d (#202) (B) for 4 d, or from a control TK-NOG mouse treated with FIAU 400 mg/kg/d for 4 d (C). The images (at 100× magnification) shown in the left column are of formalin-fixed paraffin-embedded tissues stained with a monoclonal anti–human cytokeratin 8 antibody and counterstained with hematoxylin. The antibody-stained dark brown regions have human hepatocytes, while the mouse hepatocytes appear in the light tan regions. The middle and right columns are higher magnification views (600×) of the boxed regions of the images shown on the left; the upper and lower images in (A) and (B) are from the boxed regions containing human and mouse hepatocytes, respectively. The middle column shows formalin-fixed paraffin-embedded tissues stained with H/E, and the images in the right column were stained with oil red O and counterstained with hematoxylin. The hepatocytes in livers obtained from the FIAU-treated control or from untreated mice with humanized livers do not contain the markedly increased number of vacuoles that are present in the H/E-strained sections of the FIAU-treated humanized mouse liver. Many of these vacuoles are stained with oil red O (arrows), which indicates that they contain fat. However, not all of the vacuoles are stained by oil red O (arrowheads).

**Figure 5:**
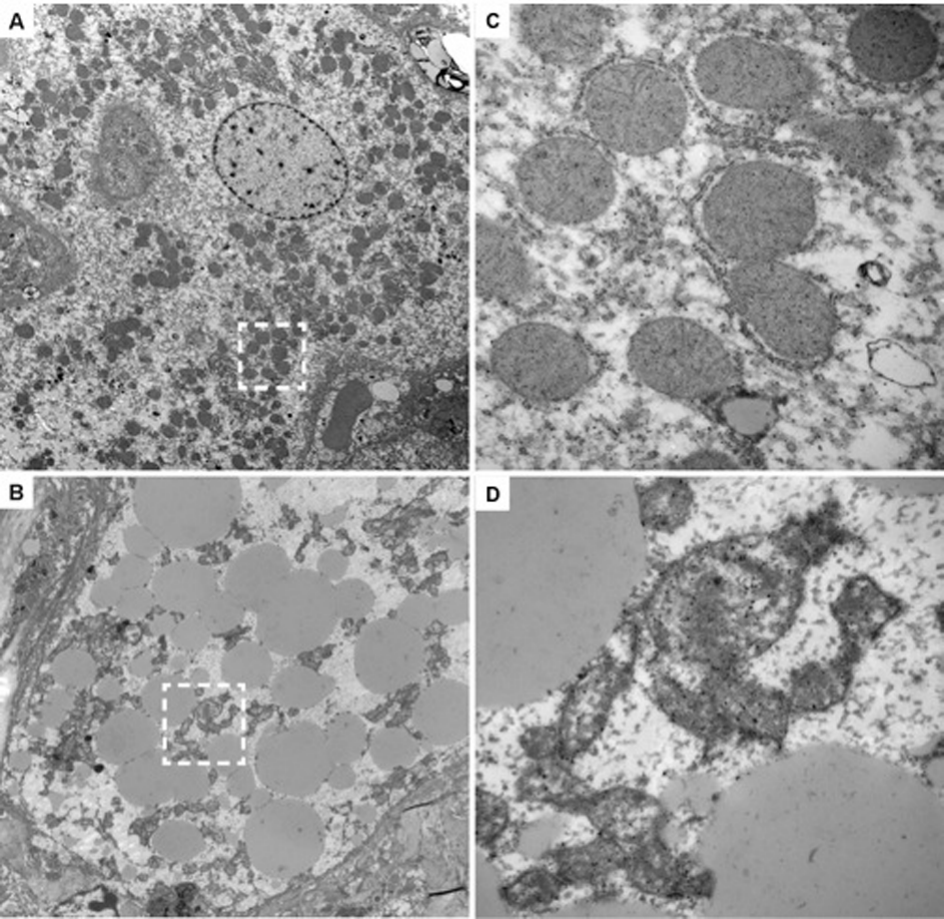
Ultra-structural changes induced by FIAU in TK-NOG mice with humanized livers. TEMs (original magnification 2,500×) of liver sections with human cells in liver tissue obtained from untreated (#202) (A) or FIAU-treated (#183) (B) chimeric mice are shown. (C) and (D) are enlarged views (30,000×) of the boxed regions in (A) and (B), respectively. The human hepatocytes within the liver tissue obtained from the FIAU-treated chimeric mouse (B) have many large lipid droplets, which are not found in the human hepatocytes in the untreated chimeric mouse (A). There are abundant mitochondria in the human hepatocytes in the untreated chimeric mouse, and cristae are readily apparent (C). In contrast, human hepatocytes in FIAU-treated chimeric mice have altered mitochondria with variable sizes and a reduced number of cristae (D).

The histology of the livers obtained from chimeric TK-NOG mice treated with FIAU 400 mg/kg/d for 4 d had abnormalities that were restricted to the regions with engrafted human liver; the regions with remnant endogenous mouse liver were identical to those in untreated chimeric TK-NOG mice (Figure 4A and 4B). The livers of FIAU-treated mice with humanized livers had extensive vacuolar changes in the regions that contained human hepatocytes, and the presence of fat in these vacuoles was confirmed by staining with oil red O (Figure 3). No abnormalities were noted in the liver sections obtained from control TK-NOG mice that were treated with FIAU 400 mg/kg/d for 14 d (Figure 4C). Although they were less pronounced, fatty changes were also present in regions with human hepatocytes in livers obtained from at least two of the four chimeric mice examined after treatment with FIAU 100 mg/kg/d for 17 d (Figure S2). However, there were no apparent changes in fat vacuole content in the livers of the mice with humanized livers treated with FIAU 2.5 mg/kg/d for 14 d (Figure S3). Ultra-structural analysis by TEM indicated that the cytoplasm of human hepatocytes in the FIAU-treated chimeric mice had many enlarged fat vacuoles and mitochondria, which were characterized by their abnormal size and shape, and by a decreased number of cristae (Figure 5). The electron micrographs confirm that FIAU treatment caused mitochondrial damage and lipid accumulation selectively in human cells within the livers of chimeric TK-NOG mice.

### Sofosbuvir Did Not Induce Liver Toxicity in TK-NOG Mice with Humanized Livers

**Figure 6:**
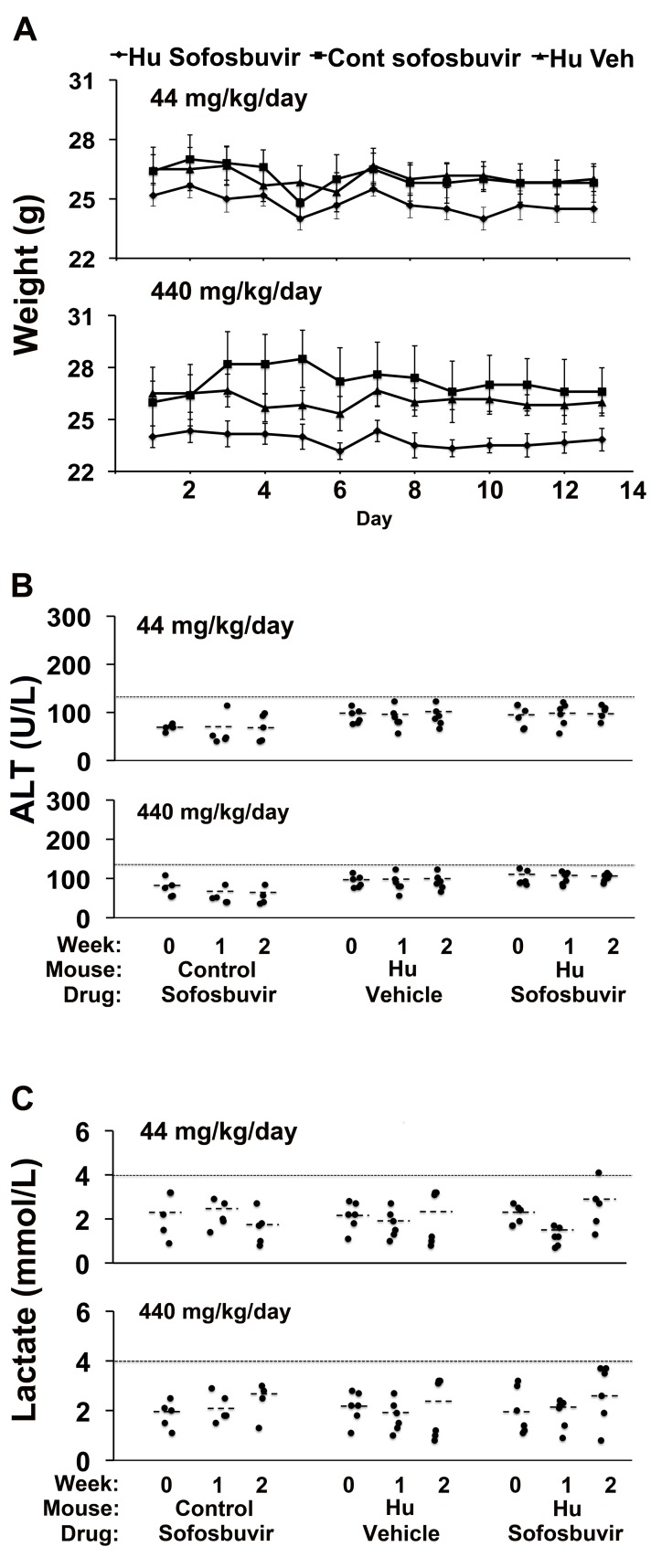
Sofosbuvir does not cause liver toxicity in TK-NOG mice with humanized livers. Control (non-humanized) (Cont, *n* = 5 per group) or chimeric TK-NOG mice (Hu, *n* = 6 per group) were treated with sofosbuvir (440 or 44 mg/kg/d po) or vehicle (Veh) for 14 d, and their body weights (A) and plasma ALT (B) and lactate (C) levels were measured on the indicated days. (A) There was no difference in the weights of vehicle- or sofosbuvir-treated control TK-NOG mice or TK-NOG mice with humanized livers. In (B and C), each symbol represents the value measured in a control TK-NOG mouse or a TK-NOG mouse with a humanized liver, the dotted line across the graph indicates the upper limit of normal, and the short dashed lines show the average for each group.

Sofosbuvir is another nucleotide analogue, used for treatment of hepatitis C virus infection, but it does not cause liver toxicity in humans [5],[6]. We examined the effect that administration of sofosbuvir 440 or 44 mg/kg/d po for 14 d had on control TK-NOG mice and TK-NOG mice with humanized livers. These murine doses are ∼90 and 9-fold, respectively, above the absolute human dose of sofosbuvir (∼5 mg/kg/d) and, after adjustment for allometric differences, correspond to approximately the current human dose and 10-fold above it. Both sofosbuvir doses were well tolerated; none of the drug-treated mice lost weight (Figure 6A), there were no deaths, and none of the drug-treated mice became overtly jaundiced. Plasma ALT and lactate levels were measured at baseline, and after 7 and 14 d of treatment. The average plasma ALT levels in mice with humanized livers in the 440- (102 U/l+4.1) and 44-mg/kg/d (100.2 U/l+6.7) treatment groups were below the upper limit of normal (120 U/l), and were not significantly different from those measured in vehicle-treated mice with humanized livers (*p* = 0.052 and 0.44 for the 440- and 44-mg/kg/d treatment groups, respectively) (Figure 6B). The plasma lactate levels were also not elevated in or control mice or mice with humanized livers receiving either dose of sofosbuvir (Figure 6C). Consistent with the laboratory data, no histopathologic changes were noted in the livers of sofosbuvir-treated mice with humanized livers (Figure S4). Thus, a nucleoside analogue that is not hepatotoxic in humans did not cause liver toxicity in TK-NOG mice with humanized livers.

## Discussion

FIAU caused the rapid onset of acute liver failure in chimeric mice. The clinical features (jaundice, lethargy), laboratory abnormalities (lactate elevation), histology, and ultra-structural changes in FIAU-treated chimeric mice mirrored those observed in FIAU-treated human participants [1]. The more rapid onset of acute liver failure in chimeric mice treated with the higher FIAU doses was the only feature that differed from that in the human participants, where a dosing period of at least 2 mo elapsed before the toxicity appeared. The more rapid onset may be due to the fact that the chimeric mice were treated with very high doses of FIAU, since the onset of the toxicity was delayed and was less severe in chimeric mice that were treated with the lower doses of FIAU. Nevertheless, FIAU-induced liver toxicity was easily detected in chimeric mice treated with a FIAU dose that was only 10-fold above that used in humans. Most importantly, drug-induced liver toxicity was not observed after mice with humanized livers were treated with sofosbuvir, a nucleoside analogue that is used for treatment of hepatitis C virus infection, which does not cause liver toxicity in human participants.

FIAU-induced toxicity occurred in mice with a severely compromised immune system that were not infected with a hepatotropic virus. This indicates that FIAU-induced toxicity is caused by a direct effect of the drug on human hepatocytes, that it does not require a co-morbidity (e.g., hepatotropic viral infection), and that it is not immune mediated. An attractive hypothesis for the mechanism of FIUA-induced toxicity is that a nucleoside transporter (equilibrative nucleoside transporter 1 [ENT1]) is expressed in the mitochondrial membranes of human, but not rodent, cells [2]. However, regardless of the specific mechanism, this study provides, to our knowledge, the first definitive example of a human-specific drug-induced toxicity that could be detected in chimeric mice, but not in conventional rodent, dog, or monkey toxicity studies [3].

Liver is the target organ for many drug-induced toxicities, and some of these are human-specific. Therefore, toxicology studies using chimeric mice could have a large impact on drug development, and could improve the safety of drugs that will be subsequently tested in humans. Moreover, we have previously demonstrated that the rate of drug metabolism in chimeric mice can be selectively regulated by transplantation of hepatocytes obtained from human donors with different alleles in genes affecting drug metabolism [12]. Since it is most often a drug metabolite, and not the parent drug itself, that is responsible for an unexpected drug-induced toxicity [16],[17], chimeric mice with human hepatocytes obtained from selected donors could be used to evaluate the potential for drug toxicities in specific vulnerable populations.

It is also important to consider the current limitations of using chimeric mice for toxicology testing. Since they are highly immunocompromised, chimeric mice cannot be used to analyze immune-mediated drug toxicities. Since FIAU exerts a direct toxic effect on hepatocytes via a specific mechanism [2], it is important to determine whether other drugs that cause human-specific toxicities by other mechanisms could be identified in chimeric mice. For example, we need to determine whether drugs causing human-specific cholestatic toxicity (i.e., bosentan [18]) can be detected using chimeric mice. To date, the vast majority of published studies using chimeric mice have examined whether they can produce known human drug metabolites [8]. We hope that the results presented here will stimulate the use of chimeric mice in preclinical toxicology studies, since the application of 21st century methodologies could improve the safety of 21st century drug development.

## Supporting Information

Checklist S1 **ARRIVE checklist.**
(DOCX)

Figure S1 **Control (non-humanized) TK-NOG mice or TK-NOG mice with humanized livers were treated with FIAU 100 mg/kg/d or vehicle for 17 d.** Left panel: The weights of the mice (average ± standard deviation) in each of the three groups (*n* = 6 mice per group) were measured daily. Chimeric (Hu) mice were heavier at the onset of the study because they were used 8 wk after transplantation of human cells, and were older than the control TK-NOG mice. Right panel: The plasma ALT was measured on day 17 for each of the indicated groups of mice. Each symbol represents the value measured in a control TK-NOG mouse or a TK-NOG mouse with a humanized liver, the dashed line across the graph indicates the upper limit of normal, and each short dashed line shows the average for each group. Only the FIAU-treated mice with humanized livers lost weight and had an increase in their plasma ALT.
(TIF)

Figure S2 **Fatty liver in FIAU-treated mice.** H/E-stained sections of liver regions containing human hepatocytes obtained from the chimeric mice (indicated by number), which received 400 mg/kg/d FIAU for 4 d, 100 mg/kg/d FIAU for 17 d, or 0 mg/kg/d FIAU. Fat vacuoles are readily apparent in two (#87 and #192) of three mice examined that received FIAU 400 mg/kg/d. Although the changes are less pronounced, fatty vacuoles are also apparent in at least two (#109 and #140) of four mice examined after treatment with FIAU 100 mg/kg/d. Arrows indicate the location of fat vacuoles. The original magnification in each panel is 600×.
(TIF)

Figure S3 **Liver histology in mice with humanized livers treated with FIAU 2.5 mg/kg/d.** H/E-stained sections of liver regions containing human hepatocytes obtained from the chimeric mice (indicated by number) that received 2.5 mg/kg/d FIAU or vehicle (0 mg/kg/d FIAU) for 14 d. A comparable number of areas with presumptive fat vacuoles (indicated by arrows) were observed in liver sections obtained from drug- and vehicle-treated mice. The original magnification in each panel is 600×.
(TIF)

Figure S4 **Liver histology in mice with humanized livers treated with sofosbuvir.** H/E-stained sections of liver regions containing human hepatocytes obtained from the chimeric mice (indicated by number) that received 44 mg/kg/d sofosbuvir, 440 mg/kg/d sofosbuvir, or vehicle (0 mg/kg/d sofosbuvir) for 14 d. A comparable number of fat vacuoles (indicated by arrows) were observed in liver sections obtained from drug- and vehicle-treated mice. The original magnification in each panel is 600×.
(TIF)

Table S1 **Chimeric TK-NOG mice used in this study.** Chimeric mice were prepared using hepatocytes obtained from six different human donors (donors 1 to 6) with the following characteristics: 3-y-old female, 2-y-old female, 10-mo-old female, 2-y-old male, 7-mo-old female, and 1-y-old female, respectively. The hepatocyte donor; FIAU (mg/kg/d), sofosbuvir (mg/kg/d), or vehicle (DMSO) dose group; and the human serum albumin level (mg/ml) are shown for each mouse used in this study.
(DOCX)

## Abbreviations

ALT: alanine aminotransferase
FIAU: fialuridine
H/E: hematoxylin and eosin
TEM: transmission electron micrograph
