# Supplementary figures and images for: Fialuridine Induces Acute Liver Failure in Chimeric TK-NOG Mice: A Model for Detecting Hepatic Drug Toxicity Prior to Human Testing
Source: PLoS Med. 2014 Apr 15;11(4):e1001628. doi: 10.1371/journal.pmed.1001628 (PMC3988005; doi:10.1371/journal.pmed.1001628)

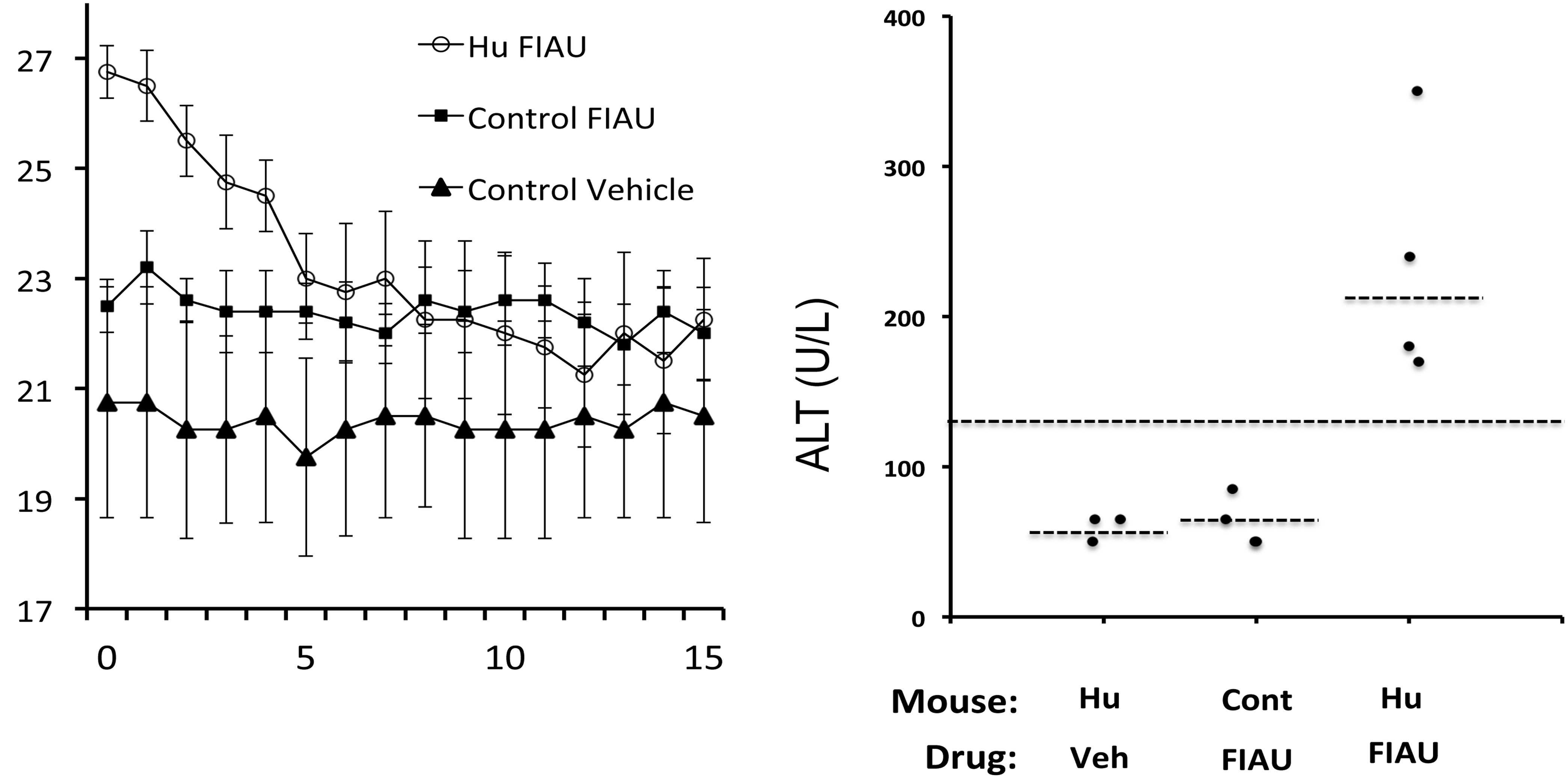

Supplement: Figure S1 — Control (non-humanized) TK-NOG mice or TK-NOG mice with humanized livers were treated with FIAU 100 mg/kg/d or vehicle for 17 d. Left panel: The weights of the mice (average ± standard deviation) in each of the three groups (n = 6 mice per group) were measured daily. Chimeric (Hu) mice were heavier at the onset of the study because they were used 8 wk after transplantation of human cells, and were older than the control TK-NOG mice. Right panel: The plasma ALT was measured on day 17 for each of the indicated groups of mice. Each symbol represents the value measured in a control TK-NOG mouse or a TK-NOG mouse with a humanized liver, the dashed line across the graph indicates the upper limit of normal, and each short dashed line shows the average for each group. Only the FIAU-treated mice with humanized livers lost weight and had an increase in their plasma ALT. (TIF) [file pmed.1001628.s002.tif]

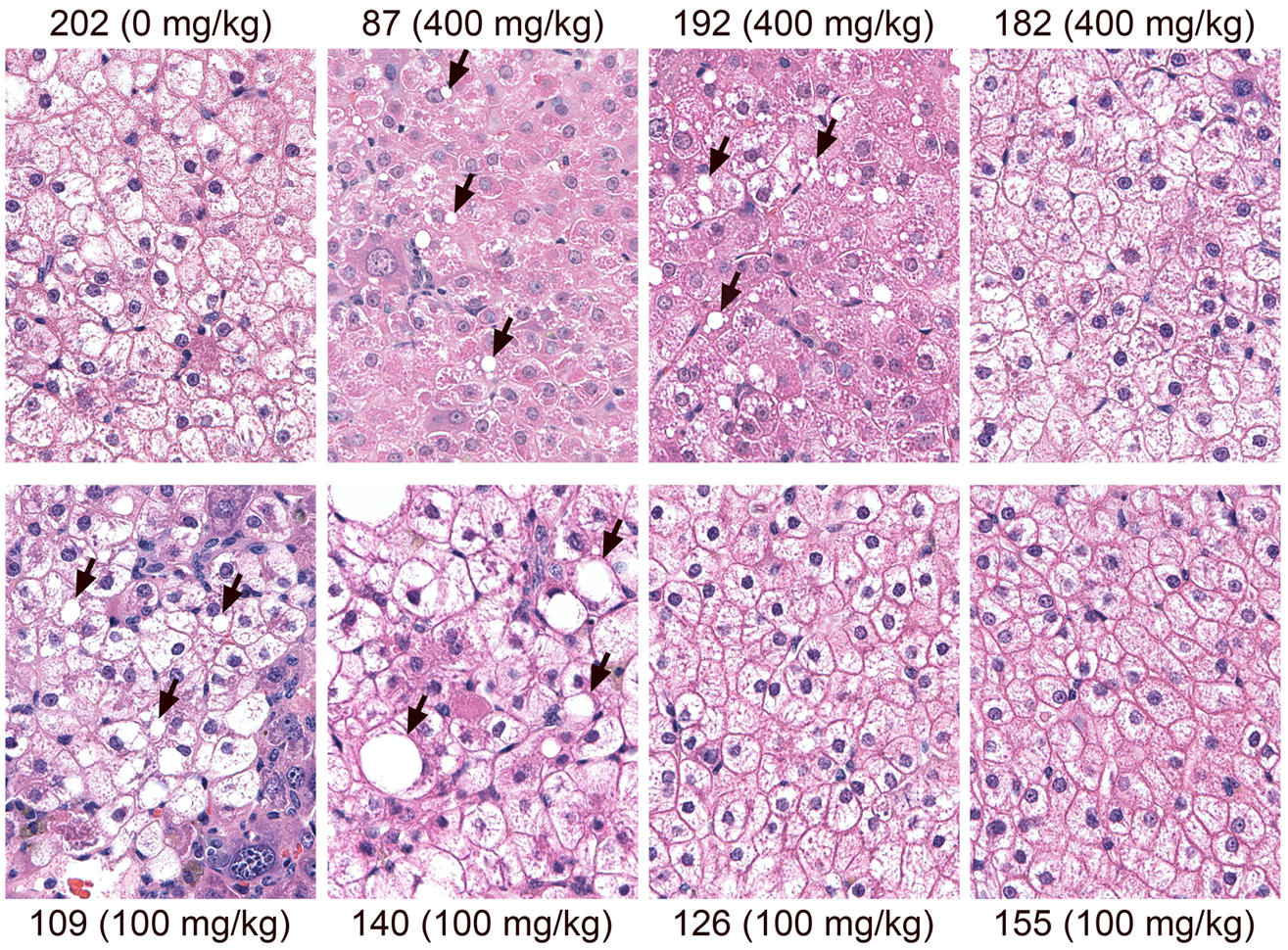

Supplement: Figure S2 — Fatty liver in FIAU-treated mice. H/E-stained sections of liver regions containing human hepatocytes obtained from the chimeric mice (indicated by number), which received 400 mg/kg/d FIAU for 4 d, 100 mg/kg/d FIAU for 17 d, or 0 mg/kg/d FIAU. Fat vacuoles are readily apparent in two (#87 and #192) of three mice examined that received FIAU 400 mg/kg/d. Although the changes are less pronounced, fatty vacuoles are also apparent in at least two (#109 and #140) of four mice examined after treatment with FIAU 100 mg/kg/d. Arrows indicate the location of fat vacuoles. The original magnification in each panel is 600×. (TIF) [file pmed.1001628.s003.tif]

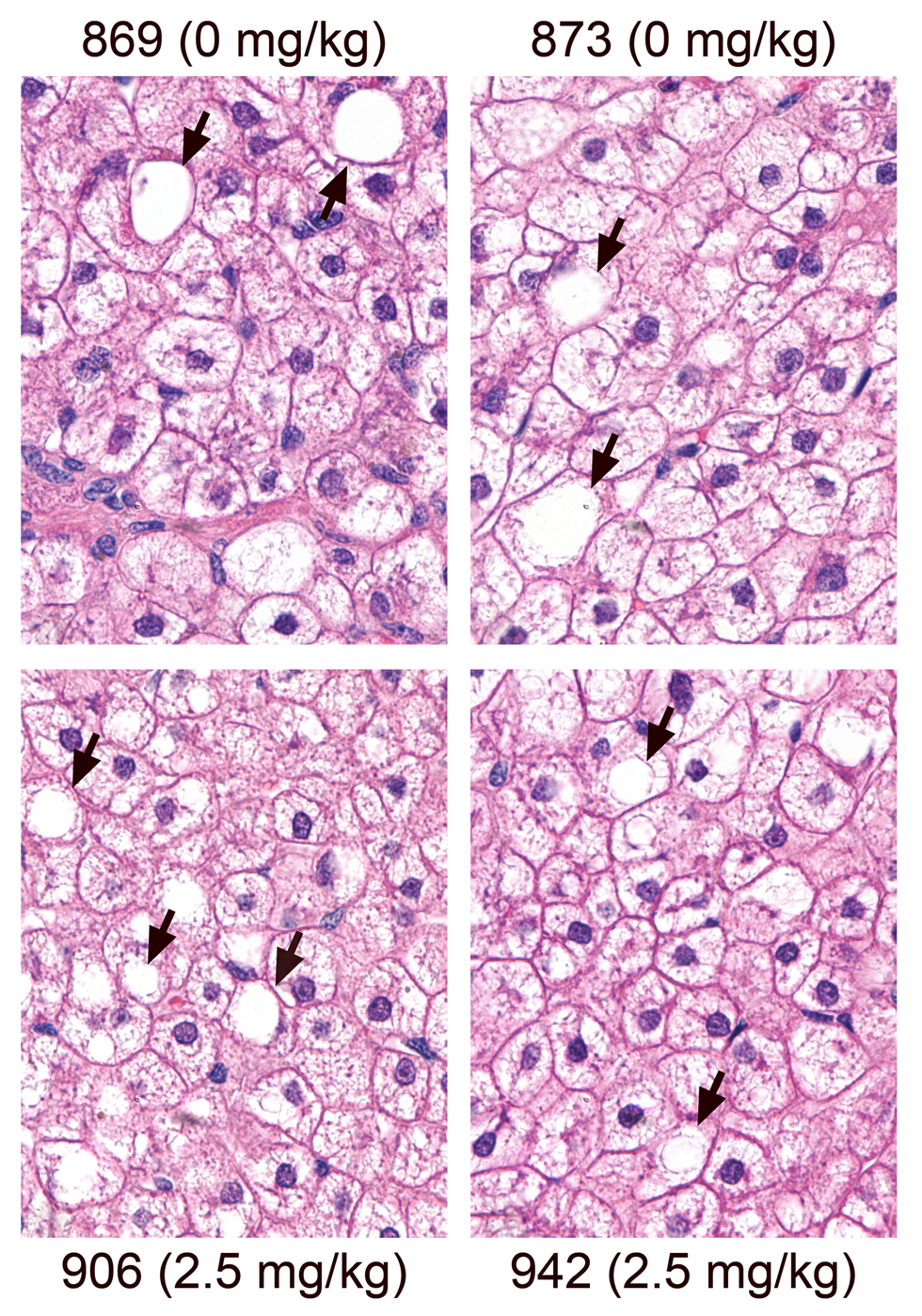

Supplement: Figure S3 — Liver histology in mice with humanized livers treated with FIAU 2.5 mg/kg/d. H/E-stained sections of liver regions containing human hepatocytes obtained from the chimeric mice (indicated by number) that received 2.5 mg/kg/d FIAU or vehicle (0 mg/kg/d FIAU) for 14 d. A comparable number of areas with presumptive fat vacuoles (indicated by arrows) were observed in liver sections obtained from drug- and vehicle-treated mice. The original magnification in each panel is 600×. (TIF) [file pmed.1001628.s004.tif]

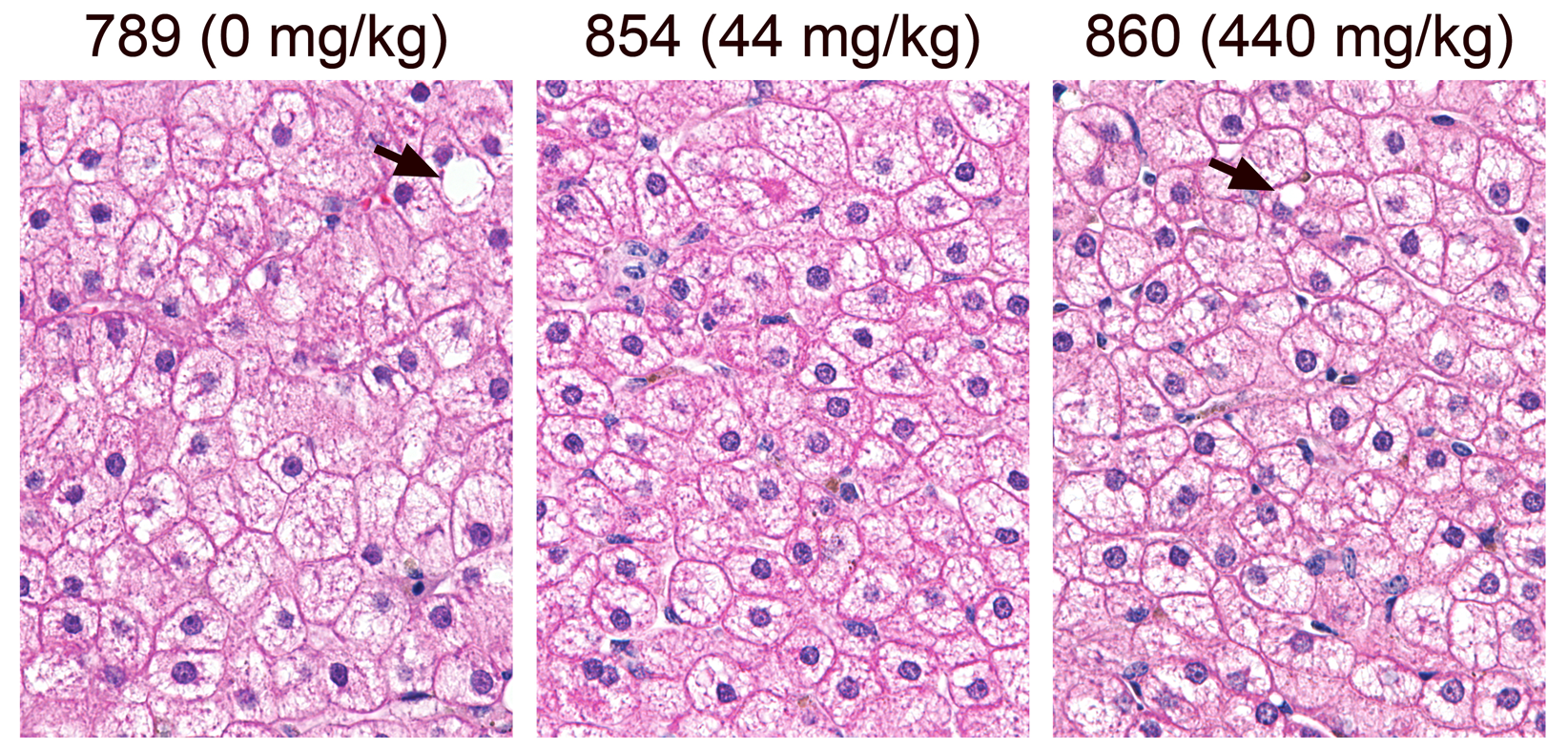

Supplement: Figure S4 — Liver histology in mice with humanized livers treated with sofosbuvir. H/E-stained sections of liver regions containing human hepatocytes obtained from the chimeric mice (indicated by number) that received 44 mg/kg/d sofosbuvir, 440 mg/kg/d sofosbuvir, or vehicle (0 mg/kg/d sofosbuvir) for 14 d. A comparable number of fat vacuoles (indicated by arrows) were observed in liver sections obtained from drug- and vehicle-treated mice. The original magnification in each panel is 600×. (TIF) [file pmed.1001628.s005.tif]
