# Supplementary material for: Fialuridine Induces Acute Liver Failure in Chimeric TK-NOG Mice: A Model for Detecting Hepatic Drug Toxicity Prior to Human Testing
Source: PLoS Med. 2014 Apr 15;11(4):e1001628. doi: 10.1371/journal.pmed.1001628 (PMC3988005; doi:10.1371/journal.pmed.1001628)
Supplement: Table S1 — Chimeric TK-NOG mice used in this study. Chimeric mice were prepared using hepatocytes obtained from six different human donors (donors 1 to 6) with the following characteristics: 3-y-old female, 2-y-old female, 10-mo-old female, 2-y-old male, 7-mo-old female, and 1-y-old female, respectively. The hepatocyte donor; FIAU (mg/kg/d), sofosbuvir (mg/kg/d), or vehicle (DMSO) dose group; and the human serum albumin level (mg/ml) are shown for each mouse used in this study. (DOCX) [file pmed.1001628.s006.docx]

**Table S1**. Chimeric TK-NOG mice used in this study. Chimeric mice were prepared using hepatocytes obtained from six different donors (donors 1 to 6) with the following characteristics: 3-year-old female, 2-year-old female, 10 month old female, 2 year old male, 7 month old female, and 1 year old female, respectively. The hepatocyte donor, FIAU (mg/kg/day), sofosbuvir or vehicle (DMSO) dose group, and the human serum albumin level (mg/ml) are shown for each mouse used in this study.

| **Mouse #** | **Donor** | **Dose (mg/kg)** | **Drug** | **Hu Alb** |
| --- | --- | --- | --- | --- |
| 64 | 1 | 400 | FIAU | 12.3 |
| 69 | 1 | 400 | FIAU | 17.4 |
| 87 | 1 | 400 | FIAU | 18.2 |
| 103 | 2 | 400 | FIAU | 10.5 |
| 81 | 2 | 400 | FIAU | 6.5 |
| 149 | 2 | 400 | FIAU | 7.8 |
| 152 | 2 | 400 | FIAU | 6.4 |
| 154 | 2 | 400 | FIAU | 16.2 |
| 161 | 2 | 400 | FIAU | 15.6 |
| 182 | 2 | 400 | FIAU | 15.1 |
| 183 | 2 | 400 | FIAU | 15.1 |
| 192 | 1 | 400 | FIAU | 11.5 |
| 196 | 1 | 400 | FIAU | 15.3 |
| 199 | 1 | 400 | FIAU | 16.7 |
| 200 | 1 | 400 | FIAU | 8.4 |
|  |  |  |  |  |
| 109 | 1 | 100 | FIAU | 7.6 |
| 140 | 1 | 100 | FIAU | 7.8 |
| 189 | 2 | 100 | FIAU | 12.4 |
| 155 | 2 | 100 | FIAU | 11.8 |
| 244 | 2 | 100 | FIAU | 11.2 |
| 126 | 1 | 100 | FIAU | 10.8 |
|  |  |  |  |  |
| 390 | 1 | 25 | FIAU | 12.5 |
| 373 | 2 | 25 | FIAU | 11 |
| 369 | 1 | 25 | FIAU | 16.5 |
| 380 | 2 | 25 | FIAU | 10.9 |
| 622 | 2 | 25 | FIAU | 7.9 |
| 633 | 2 | 25 | FIAU | 18.2 |
|  |  |  |  |  |
| 904 | 2 | 2.5 | FIAU | 12.3 |
| 871 | 1 | 2.5 | FIAU | 13.8 |
| 906 | 2 | 2.5 | FIAU | 11.2 |
| 912 | 2 | 2.5 | FIAU | 10.5 |
| 943 | 1 | 2.5 | FIAU | 13.1 |
| 942 | 1 | 2.5 | FIAU | 14.3 |
|  |  |  |  |  |
| 202 | 2 | 0 | histology control | 9.2 |
|  |  |  |  |  |
| 390 | 2 | 5% | DMSO | 12.5 |
| 369 | 2 | 5% | DMSO | 16.5 |
| 380 | 2 | 5% | DMSO | 10.9 |
| 621 | 2 | 5% | DMSO | 14.5 |
| 620 | 1 | 5% | DMSO | 10.9 |
| 662 | 2 | 5% | DMSO | 7.8 |
|  |  |  |  |  |
| 782 | 2 | 5% | DMSO | 9.8 |
| 869 | 1 | 5% | DMSO | 10.6 |
| 867 | 1 | 5% | DMSO | 11.6 |
| 873 | 1 | 5% | DMSO | 8.8 |
|  |  |  |  |  |
| 857 | 1 | 440 | Sofosbuvir | 9.6 |
| 858 | 1 | 440 | Sofosbuvir | 10 |
| 796 | 1 | 440 | Sofosbuvir | 10.8 |
| 860 | 1 | 440 | Sofosbuvir | 13.2 |
| 831 | 1 | 440 | Sofosbuvir | 8.6 |
| 829 | 1 | 440 | Sofosbuvir | 8.8 |
|  |  |  |  |  |
| 891 | 3 | 44 | Sofosbuvir | 12.7 |
| 890 | 3 | 44 | Sofosbuvir | 12.2 |
| 897 | 3 | 44 | Sofosbuvir | 7.5 |
| 852 | 3 | 44 | Sofosbuvir | 12.5 |
| 854 | 3 | 44 | Sofosbuvir | 7.5 |
| 122 | 4 | 44 | Sofosbuvir | 15.2 |
|  |  |  |  |  |
| 69 | 5 | 5% | DMSO | 6.5 |
| 70 | 5 | 5% | DMSO | 10.6 |
| 117 | 4 | 5% | DMSO | 10 |
| 789 | 2 | 5% | DMSO | 9.8 |
| 791 | 2 | 5% | DMSO | 10.4 |
| 242 | 6 | 5% | DMSO | 8.9 |
